# Braithwaite’s Retrospect

**Published:** 1860-05

**Authors:** 


*Braithwaite's Retrospect of Practical Medicine and Surgery.* Part the Fortieth. New York: W. A. Townsend & Co.

This reprint, for January, was timely placed upon our table by the publishers. Its *three hundred and sixty pages* are filled with choicest selections from trans-Atlantic Journals. In addition to three or four American journals, every physician who intends to keep himself thoroughly informed in his profession, will take at least one of the reprints of foreign journals.

